# Reflections on liver transplantation: emerging research and clinical perspectives in 2025

**DOI:** 10.3389/frtra.2026.1923144

**Published:** 2026-08-26

**Authors:** Alfred Wei-Chieh Kow

**Affiliations:** 1 Division of Hepatobiliary & Pancreatic Surgery, Department of Surgery, National University Hospital, Singapore, Singapore; 2 Yong Loo Lin School of Medicine, National University of Singapore, Singapore, Singapore; 3 National University Centre for Organ Transplantation (NUCOT), National University Health System, Singapore, Singapore

**Keywords:** artificial intelligence, basic science, donation after circulatory death, ILTS 2025 Singapore, liver transplantation, machine perfusion, precision medicine, robotic surgery

## Abstract

The 30th Annual Congress of the International Liver Transplantation Society (ILTS), held in Singapore from 28 to 31 May 2025 under the theme “Advancing Liver Transplantation: Embracing Innovation for the Future,” was a landmark meeting—the first ILTS Congress in Southeast Asia and the largest in the Society's history, convening more than 1,200 delegates and 262 invited speakers from 40 countries across 92 scientific sessions and 7 pre-congress workshops. The congress was led by the National University Centre for Organ Transplantation (NUCOT) at the National University Hospital (NUH), with the author serving as Local Chair. This article provides a reflection on the emerging research and clinical perspectives as presented and discussed at the ILTS Annual Congress 2025 in Singapore. We also share the overall organization and experience of the meeting. This is a structured overview of the congress includes its programme architecture, main scientific findings across all subspecialty domains and the legacy Tree of Life project. There is also an in-depth thematic review of the most impactful scientific contributions, with particular attention to robotic and minimally invasive surgery, machine perfusion and donation after circulatory death (DCD) liver transplantation, transplant oncology, artificial intelligence (AI) and precision medicine, and basic and translational science. Collectively, these themes reflect a field at a genuine inflection point, where multiple technological revolutions are converging at clinical maturity whilst enduring challenges of organ scarcity, global equity, and cost sustainability demand continued attention.

## An overview of the ILTS annual congress 2025 in Singapore

The 30th Annual Congress of the ILTS marked three milestones simultaneously: the Society's three-decade anniversary (physical and virtual), its inaugural meeting in Southeast Asia and also the 10th Anniversary of The Liver Transplantation Symposium by the NUCOT at NUH. Held at Suntec Singapore, the congress was led by the NUCOT at NUH, with support from the SingHealth Duke-NUS Liver Transplant Centre and Mount Elizabeth Hospital. The opening ceremony was officiated by Professor Kenneth Mak, Director-General of Health, Ministry of Health, Singapore—reflecting the significance of the event to the national and regional health landscape.

Singapore's hosting of the congress was appropriate. The city-state's transplant programme exemplifies both the achievements and the enduring challenges of the Asia-Pacific region: technical excellence encompassing deceased and living donor liver transplantation, ABO-incompatible transplantation, and increasingly minimally invasive liver donor surgery, within a healthcare system that navigates the realities of an organ shortage common across the region. More than 400 patients remain on Singapore's national waiting list (with second largest organs needed being the liver)—a statistic that grounds the scientific ambition of the congress in immediate clinical urgency (1).

**Figure 1:**
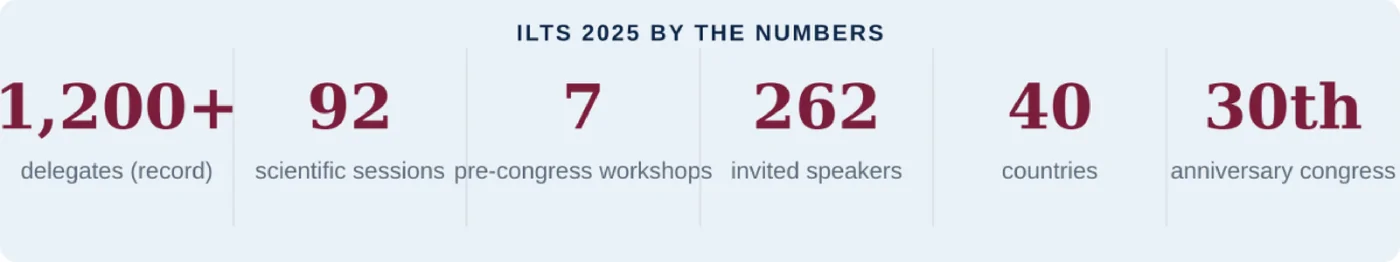
Basic statistics of the ILTS annual congress 2025 in Singapore.

The congress drew a record attendance of more than 1,200 delegates from across Asia-Pacific, Europe, the Americas, and the Middle East, along with 262 invited speakers from 40 countries [Figure 1]. The congress was held with high level scientific content with a packed programme of 92 scientific sessions and 7 pre-congress workshops. The Pre-congress workshops include the high-fidelity Simulation for Anaesthesia in Liver Transplant (SALT) with Transoesophageal Echocardiography (TEE) workshop, Machine Perfusion Workshop, Allied Health Professional Workshop, Premeeting Symposium, Business & Financial Practice Workshop, Post-graduate Course and THINK Hepatology networking session.

## Symbol of ILTS 2025 in Singapore

**Figure 2:**
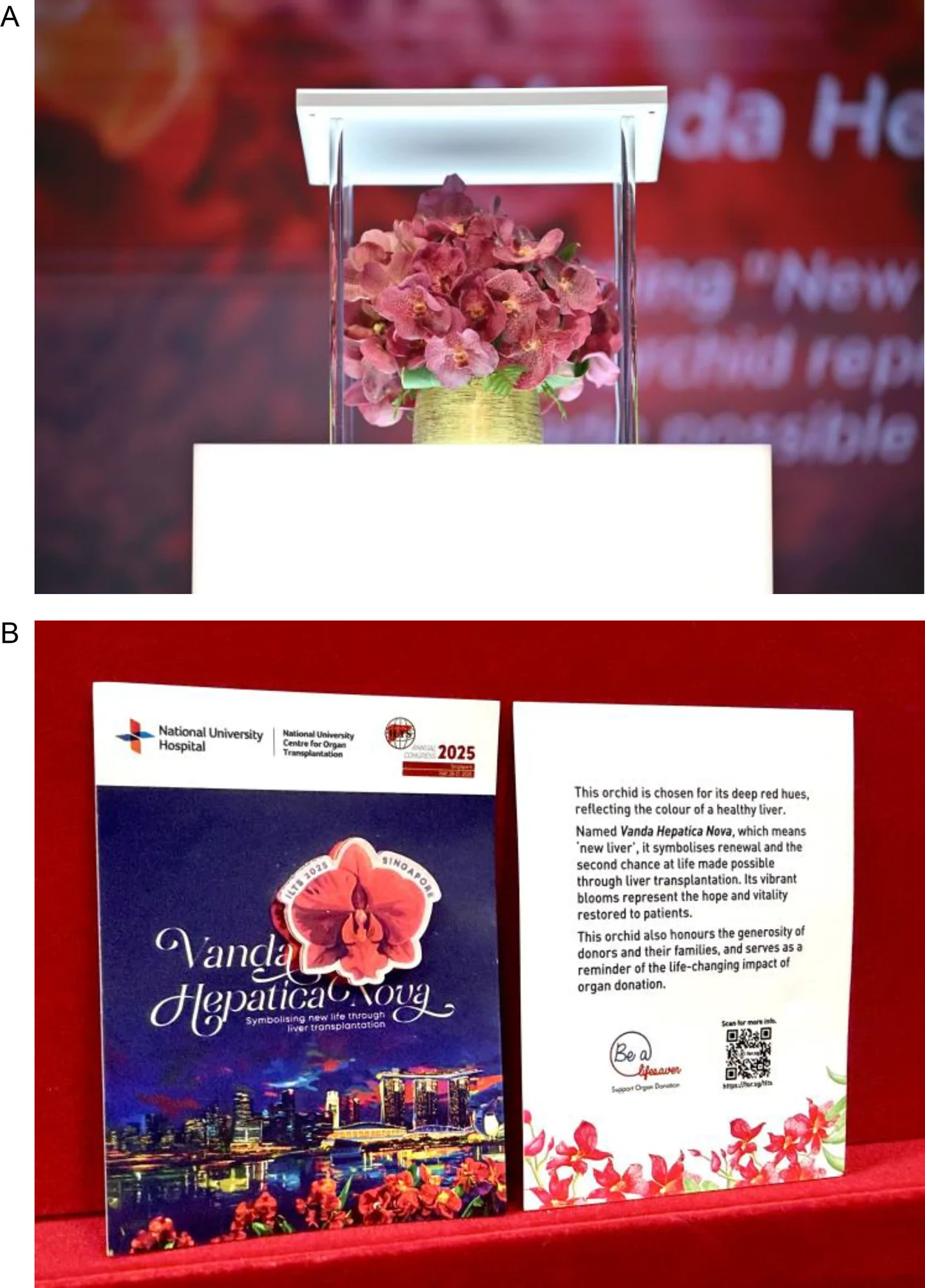
**(A)** the vanda Hepatica Nova, a new orchid breed specially grown by the botanic gardens in Singapore to commemorate the ILTS 2025 in Singapore. It symbolizes a new lease of life, as represented by its name, in Latin it means Orchid of a New Liver. **(B)** The local organizer also prepared a collar-pin with the symbol of this orchid as souvenirs for all the participants at this congress.

In a symbolic tribute to the congress, a new orchid hybrid—Vanda Hepatica Nova (Latin for “new liver”)—was launched and named in its honor, symbolizing renewal and life after transplantation, and paying tribute to donors, their families, and recipients worldwide [Figures 2A,B].

## Tree of life: A legacy beyond the meeting

**Figure 3:**
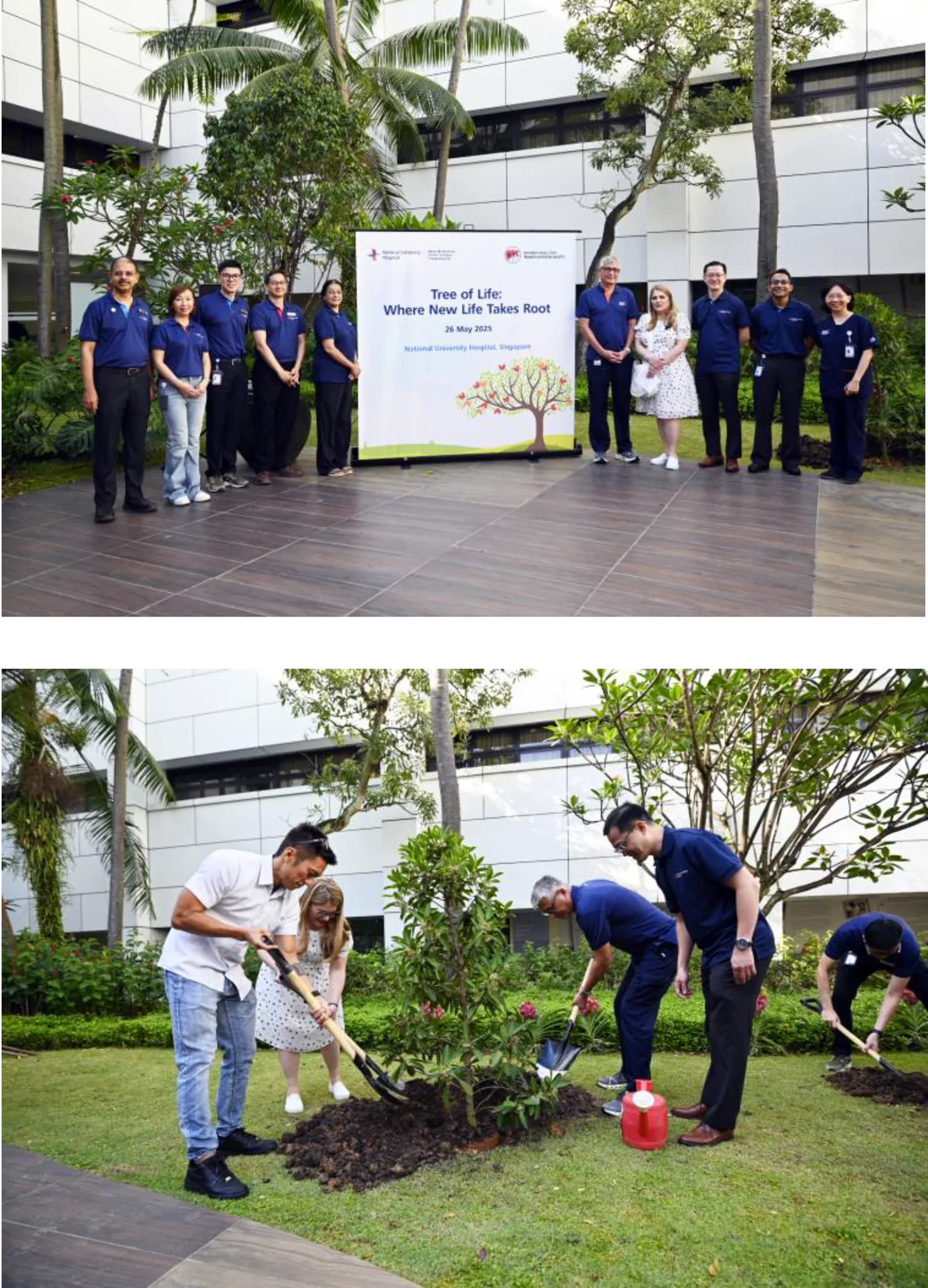
The ILTS 2025 in Singapore was marked by a tree planting legacy project called “Tree of Life: Where New Life Takes Root”.

In a meaningful prelude to the congress, NUCOT launched the inaugural Tree Planting Legacy Project—“Tree of Life: Where New Life Takes Root”—on 26 May 2025 at NUH (Figures 3A,B), with further plantings at Ng Teng Fong General Hospital, Alexandra Hospital, and Woodlands Health in Singapore. A collective gesture honouring organ donors and their families, the project was to commemorate the invaluable sacrifices made by organ donors and to raise awareness about the life-changing impact of organ donation. Organ donation helps to save lives and gives patients with end-stage organ disease a chance to have a new lease of life.

Each tree planted will symbolise a living tribute to remind us of the precious gift of life and renewal made possible through organ transplantation. Just as a tree grows strong from its roots, the gift of organ donation creates ripples of hope, renewal and gratitude—changing the lives for generations to come. It tied the global congress to a deeply local truth: that the science of transplantation is only ever possible because with organ donations from the donors and also the commitment of the healthcare professionals to work hand in hand to help the recipients.

## Program architecture

The Scientific Programming Committee—chaired by ILTS President-Elect Professor Valeria Mas, with Associate Professor Alfred Kow as Local Chair, Adjunct Associate Professor Mark Muthiah as Local Co-Chair and Professor Nazia Selzner as the ILTS President—redesigned the congress programme around three deliberate axes: deeper subspecialty content, broader allied-health involvement, and stronger Asia-Pacific representation. As per the principle of collaboration and attracting the best suggestions from the wider liver transplant community that are actively involved in the ILTS, the scientific programming committee accepted proposals from all the committees and Special Interest Groups (SIGs) in the ILTS as well as working closely with other societies and association (for the joint sessions) to formulate the final high quality scientific programs for the ILTS Annual Congress 2025. In addition, speakers for SOTA lectures were specially and carefully curated from experts in the field by the scientific committee.

Seven pre-congress workshops (28 May 2025) set the tone:

- Hands-on liver machine perfusion (HOPE and NMP) with live demonstrations represented by all major devices in the machine perfusion arena
- High-fidelity Simulation for Anaesthesia in Liver Transplant (SALT) with Transoesophageal Echocardiography (TEE) workshop with case-based panels
- Business & Financial Practice workshop
- Allied Health Professionals (AHP) workshop—a new track recognising the central role of transplant coordinators, nurses, pharmacists, and rehabilitation teams
- Premeeting Symposium focusing on:
- Advances in Cirrhosis, Acute Kidney Injury and Liver Transplantation—Updated Definitions, Pre-Transplant Strategies, and Allocation Policy
- Getting patients with Colorectal Liver Metastasis or Intrahepatic Cholangiocarcinoma ready for liver transplantation in Real World: Work-up and State of the Art Neoadjuvant Therapy
- MASH and Transplantation—Emerging Therapies, Challenges, and Opportunities
- Post-graduate Course for trainees
- THINK Hepatology—Liver transplant hepatologist networking session

The three-day main programme (29–31 May 2025) comprised state-of-the-art lectures, plenary panels, subspecialty tracks (surgery; anaesthesia and critical care; transplant oncology; transplant infectious diseases; immunology and basic science; paediatric transplantation), industry symposia and innovation showcases, meet the expert, oral and flash posters abstract and late-breaking abstract sessions.

## Main scientific findings: emerging research and clinical perspectives

**Figure 4:**
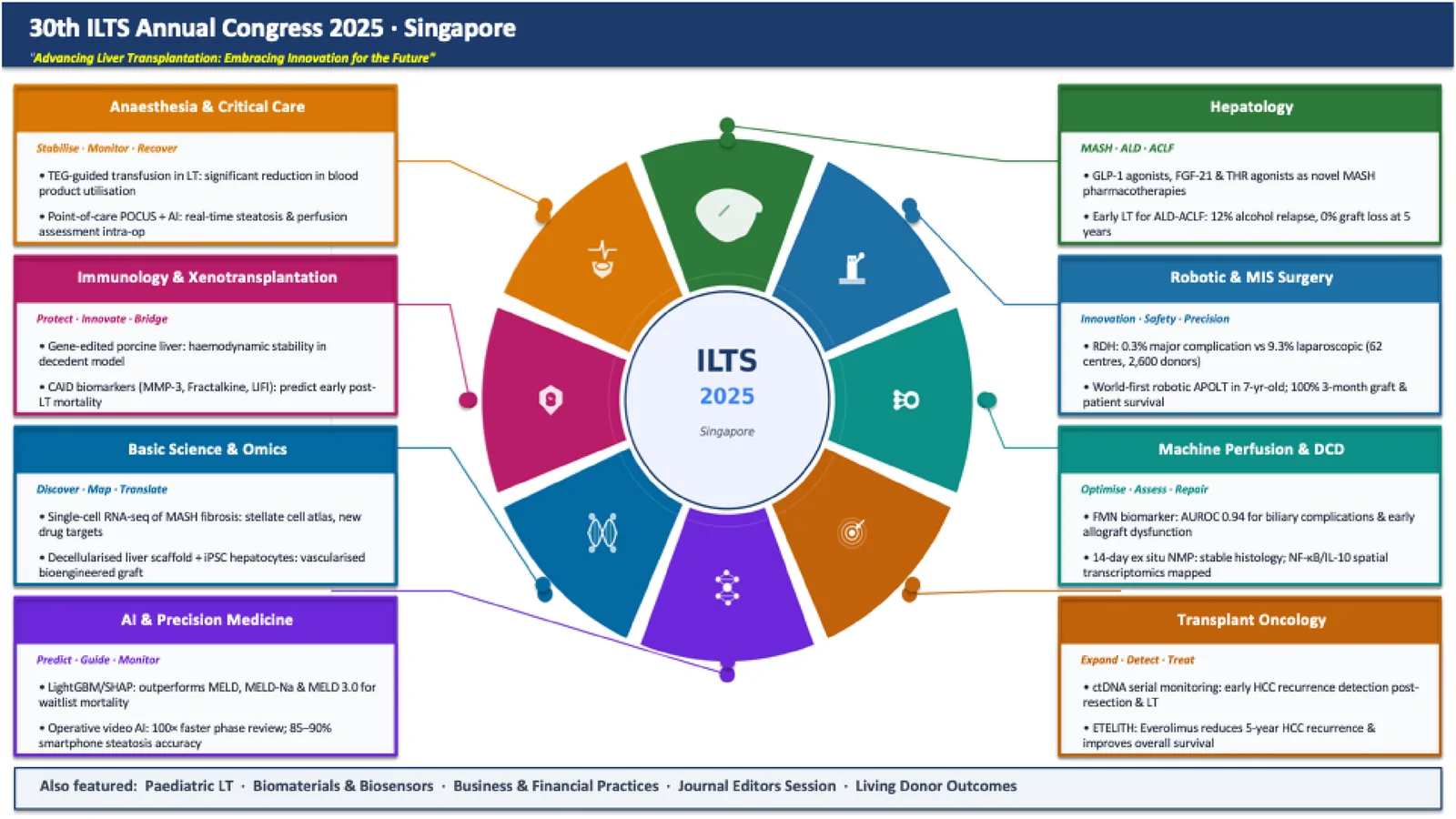
Summary for major sessions at the ILTS annual congress 2025—illustrating the current hot topics in the field of liver transplantation worldwide.

The scientific programme of the 30th ILTS Annual Congress attracted a record scientific submission pool of 794 abstracts, with 262 oral, plenary, flash, and rising star presentations drawn from a global pool spanning 40 countries. This section provides a thematic in-depth review of the most significant scientific contributions across the principal domains of the congress. Abstract identifiers are cited as assigned at the congress, including RS stands for Rising Star, PA stands for Plenary Abstract, O stands for Oral abstract and FP stands for Flash Poster (Figure 4).

## State of the art lectures (SOTA)

There were 3 State-of-The-Art lectures at the ILTS Annual Congress 2025 in Singapore. The topics encompassed the latest advances in healthcare and organ transplantation. Professor Dean Ho from the National University of Singapore spoke on “AI-Driven Precision Medicine: Transforming Liver Transplant Decision-Making and Patient Care”. He even shared his own personal experience of using himself as the subject for precision management of dietary intake in relation to changes in his body metabolites during the session. The second SOTA from the USA, Professor Jamil R Azzi spoke on “Novel Nanotechnology approach to overcome limitations of systemic IL-2 in tolerance”. He illustrated the application of nanotechnology to enhance immune-tolerance in organ transplantation. Lastly, Professor Mohamed Rela shared on the application of robotic technology in living donor liver transplantation. His SOTA talk on “Pushing the Boundaries with Innovative Robotic Technologies in Liver Transplantation: A Focus on Pediatric Applications” shared regarding his center's experience in using robotic platform to perform paediatric LDLT in India. The SOTA lectures had enriched the academic advances in the field of liver transplantation.

## Innovations and safety in living donor liver transplantation

Robotic donor hepatectomy (RDH) emerged as the dominant surgical narrative of ILTS 2025. The LDLTregistry.org Collaborative analysed 2,600 donors at 62 centres and found RDH achieved the lowest blood loss, shortest hospital stay, and lowest major complication rate (0.3%) compared with laparoscopic (3.7%) and open (1.9%) approaches [O-5446] (2). A single-centre analysis of 339 living left-lobe donors reported significantly lower blood loss with RDH [77 (SD 68) vs. 316 (168) mL, *p* < 0.001] and associated lower recipient morbidity rate (40% vs. 59%, *p* = 0.033) [O-4883]. The A2ROBOT study confirmed safety across 88 RDHs at 4 US centres with 5.7% major morbidity and no donor mortality [O-4530].

Risk stratification was emphasised: portal vein complications (1.9% of right-lobe hepatectomies) were predicted by main-to-left PV angle <60°, variant anatomy, absent falciform fixation, and BMI > 30 [FP-4842]. Intraoperative hepatic artery flow < 55 mL/min was strongly associated with small-for-size syndrome [FP-5326]. The FLAME model achieved AUC 0.81 for early graft failure using four postoperative markers [O-5023]. In an open-label RCT conducted to study methods to enhance liver donor regeneration, ursodeoxycholic acid supplementation was found to enhance liver growth velocity by day 14 in right-lobe donors [O-4626].

## Emerging surgical techniques: minimally invasive liver transplantation

Minimally invasive LT in recipients is gaining traction. Dokmak et al. reported laparoscopic-assisted LT (LALT) in 15 patients with HCC and severe portal hypertension: operative time ∼5.5 h, anhepatic phase 57 min, no transfusion requirement, discharge day 11 [O-5251]. Robotic LT demonstrated decreased blood loss, fewer infections, shorter ICU and hospital stays, and 100% graft and patient survival at 3 months [PA-4909]. Magistri et al. (Modena) reported 7 patients with no high-grade complications or readmissions at 30 days [O-5164]. VR-based surgical training using Oculus Quest 2 with 3D liver models improved anatomical comprehension in trainees [RS-4532]. ICG fluorescence via BOAI enabled precise biliary visualisation during laparoscopic hemi-hepatectomy [O-4230] and *ex vivo* graft splitting [O-4866] (3).

## Emerging strategies in transplant hepatology: MASH, ALD & ACLF

LT is increasingly performed for MASH, ALD, and ACLF. MASH is a growing waitlist contributor with ∼20% post-LT prevalence; GLP-1 receptor agonists [O-5494], THR agonists, and FGF-21 analogues represent emerging therapeutic options. ACLF accounts for approximately one-third of transplants at high-volume centres; plasma exchange shows the most favourable bridging survival data. The Toronto group demonstrated early LDLT for ALD-ACLF without a strict 6-month abstinence rule achieves low relapse (12%) with no graft loss attributable to alcohol at 5 years when multidisciplinary evaluation is enforced [FP-5108]. The UCLA experience highlighted the central role of advanced practice providers in integrated addiction medicine teams to reduce recidivism [FP-4627].

## Anaesthesia and critical care

Wide-ranging perspectives on perioperative care were presented. Cucco et al. evaluated a modified CAD-LT algorithm for coronary revascularisation prior to LT [O-4624]. Cirrhotic cardiomyopathy showed minimal intraoperative impact but potential association with first-year heart failure [PA-4373, FP-4638]. Frailty and nutrition optimisation improved conditioning but with unclear mortality benefit [O-5327, O-4322]. Pain analysis identified female gender, bilateral subcostal incision, MELD 10–15, BMI > 30, and MASH as predictors of higher pain scores [FP-5277]. Updated ILTS-endorsed porto-pulmonary hypertension guidelines and contemporary ALF management—including plasma exchange, CRRT, and auxiliary LT—were reviewed (4).

## Machine perfusion: current state and future directions

Machine perfusion was a dominant theme of ILTS 2025 Singapore. Expert perspectives covered biliary perfusion biomarkers—low biliary glucose, high bicarbonate, and alkalotic pH correlating with viable cholangiocytes—and successful transplantation of grafts perfused up to 14 days. Caution with severe (>30%) macrovesicular steatosis even after NMP was emphasised. Future directions in graft repair included genetic editing, viral resistance strategies, and surgical modification during perfusion.

Perfusate flavin mononucleotide (FMN) emerged as a landmark biomarker. De Goeij et al. demonstrated FMN during abdominal normothermic regional perfusion (NRP) predicted biliary complications (AUROC 0.799 perfusate; 0.732 bile) [O-5294]. Cleveland Clinic data from >1,700 NMP samples validated FMN's predictive power with AUROC 0.94 over 0–4 h [PA-4467]. Prospective FMN assessment was independently associated with 44% reduction in 90-day comprehensive complication index [PA-4467]. Al-Adra et al. applied spatial transcriptomics to DCD livers before and after NMP, identifying NF-*κ*B/IL-10 downregulation as a protective signature against ischaemic cholangiopathy [PA-5361]. The Royal Prince Alfred group reported the first transcriptomic analysis of human livers maintained up to 3 weeks on long-term NMP, demonstrating functional recovery, stable metabolism, and preserved histology [PA-5126] (3).

## Transplant oncology: expanding indications

Transplant oncology continued to expand. Global leaders discussed surgical innovations beyond conventional limits: LT techniques applied to advanced perihilar cholangiocarcinoma (Prof. Pal Dag Line), the RAPIDE procedure for biliary malignancies (Prof. Deniz Balci), and ex situ colorectal liver metastasis resection (Dr Fernandez, Brazil). Dr Olivier Scatton provided a detailed overview of LT as a gold standard for rare hepatic malignancies, with excellent outcomes in neuroendocrine tumours and hepatic adenomas, but poorer results in fibrolamellar carcinoma and sarcomas.

**Figure 5:**
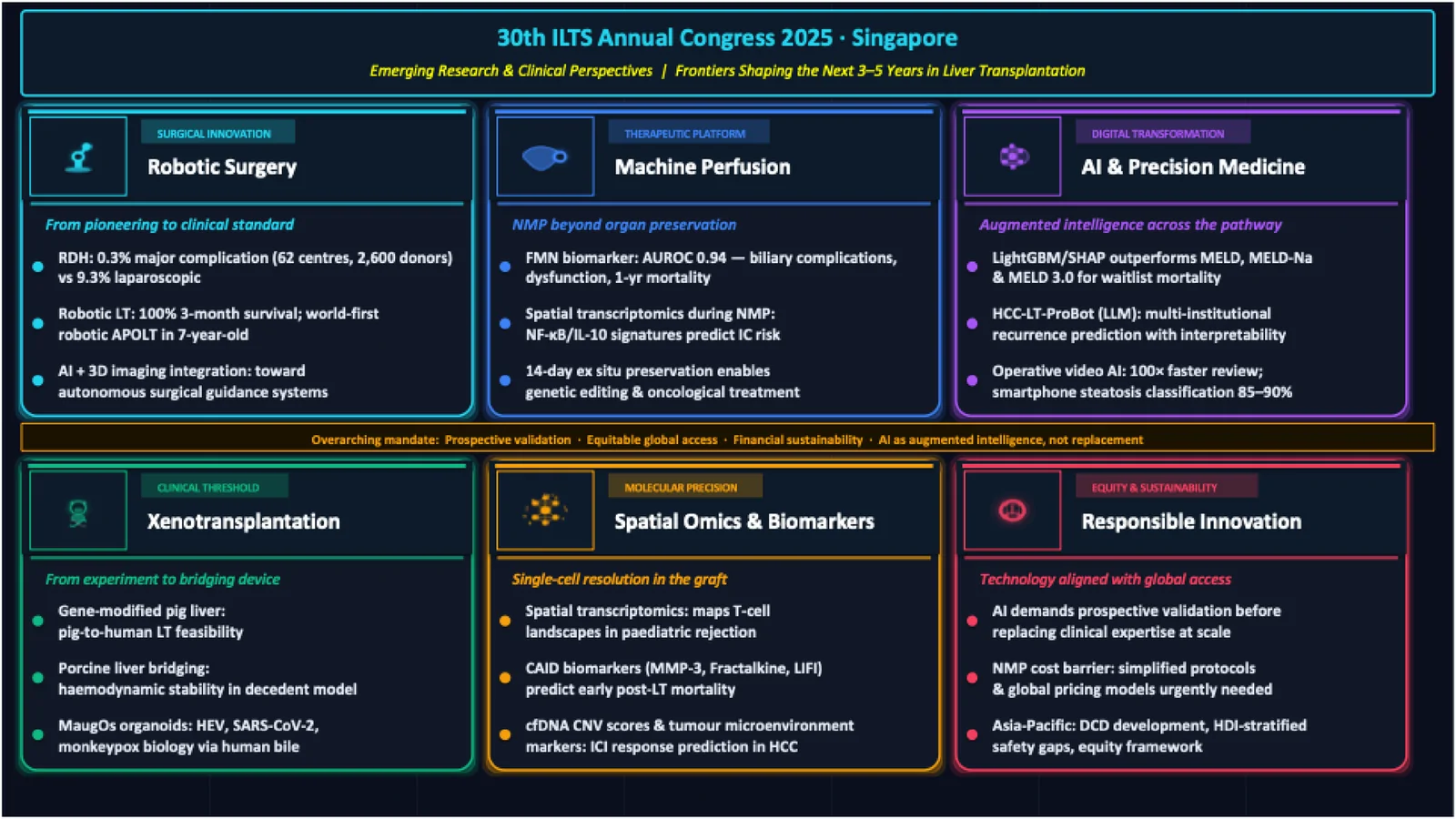
Emerging research and clinical perspectives in liver transplantation in 2025.

Abdelrahim et al. reported ctDNA serial testing effectively identifies early HCC recurrence after resection and LT [O-5100]. [Figure 5] Kounis et al. (7,981 recipients, French national data) demonstrated everolimus exhibited a protective effect against *de novo* malignancies [O-4420]. The ETELITH study demonstrated everolimus significantly reduced HCC recurrence within the first 5 years post-LT and improved overall survival [FP-4419] (3).

## Artificial intelligence and precision medicine

AI and precision medicine are transforming LT across the clinical pathway. Explainable machine learning (LightGBM with SHAP) achieved superior discrimination for short-term waitlist mortality vs. MELD, MELD-Na, and MELD 3.0, highlighting functional status, bilirubin kinetics, and ascites severity as novel predictors [RS-4418]. Automated nnU-Net segmentation demonstrated strong accuracy for HCC lesions > 2 cm [FP-4453]. Deep learning-based intraoperative navigation showed feasibility for real-time avascular-plane segmentation in MIS donor surgery [FP-5318]. HCC-LT-ProBot—an LLM integrating literature knowledge with multi-institutional datasets—predicted recurrence with high accuracy [O-4254]. Machine learning Random Forest classifiers demonstrated promising accuracy for predicting T-cell-mediated rejection non-invasively [FP-5223]. AI auto-segmentation reduced expert operative video review time ∼100-fold [FP-4935]. Smartphone photograph recognition classified donor steatosis at 85%–90% accuracy [PA-4755]. SOTA Professor Dean Ho emphasised throughout that AI is best positioned as an adjunct to—not a substitute for—clinical expertise, requiring rigorous prospective validation (3).

## Immunology, infection & xenotransplantation

In experimental xenotransplantation, building on Tao et al.'s findings on heterotopic auxillary transplantation of a 6-gene-edited porcine liver intoa brain dead human donor (5), Shaked et al. demonstrated feasibility of genetically modified porcine livers as a bridge to transplantation: haemodynamic stability, metabolic function, modulated immune activation, and reduced hyperacute rejection in a brain-dead human decedent model (6). Van der Laan's group developed macrophage-augmented organoids (MaugOs) from pluripotent stem cells, yielding insights into HEV replication via human bile, and SARS-CoV-2 and monkeypox biology (7). Dr Juliet Emamaullee reported spatial transcriptomics in paediatric ALF linked impaired T-cell exhaustion and macrophage polarisation to reduced liver regeneration. CAID biomarkers—matrix metalloproteinase-3, Fractalkine, and the Liver Immune Frailty Index—identify patients at higher early mortality risk. Infectious disease updates supported discontinuation of long-term HBIG in selected HBV-HDV co-infection and vigilance for MDR Enterobacterales.

## Paediatric liver transplantation

Paediatric sessions focused on robotics, surgical technique, immunological challenges, and perioperative care. Professor Mohamed Rela's world-first robotic APOLT in a 7-year-old child with a urea cycle defect demonstrated precision, faster recovery, reduced scarring, and minimised psychological impact—recognised as a transformative step in paediatric LT. Portal vein reconstruction strategies for veins < 5 mm in biliary atresia were detailed by Dr Kasahara (Japan) and Dr Sayed (Canada). Lee et al. presented a systematic review of ABO-incompatible paediatric LT in the rituximab era: 1-year graft survival 99.3%, patient survival 97% [O-4820]. ROTEM-guided transfusion significantly reduced blood product use [O-4439].

## Financial and business practices in liver transplantation

The Business Practice Symposium highlighted the financial and strategic implications of innovation. Dr Ghinolfi (Italy) underscored the high costs of combined perfusion strategies, calling for simplification, training, and global pricing models to expand access. Dr Zarrinpar (USA) presented AI as “augmented intelligence” with applications in donor matching, risk prediction, and immunosuppression, emphasising transparency and responsible integration. Dr Martin Dib (USA) addressed robotic transplantation economics, weighing startup costs against long-term efficiencies and proposing novel funding models. Dr Daniel Maluf (USA) provided a macroeconomic perspective on ageing populations, chronic disease burden, and the future role of AI, gene therapy, and organ repair. The panel collectively called for aligning innovation with sustainability, equity, and evidence to ensure a financially viable future for LT.

## Interesting and new sessions at the ILTS annual congress 2025

### Biomaterials, biosensors, and advances in basic science

Next-generation biomaterials for organ preservation and bile-duct reconstruction, continuous real-time graft monitoring sensors during *ex vivo* perfusion, and wearable platforms for post-transplant complication detection represented emerging technologies near clinical application. In the session on “Next-Gen Solutions: Biomaterials and Biosensors Shaping the Future of Liver Disease and Transplantation”, various topics including designing nanopaste biomaterials to eliminate cancer: Current Applications and Future Perspectives, Leveraging Perfusion Pump Systems for Mitochondrial Therapy and Defatting in Liver Steatosis, Cholangiocyte senescence—How to identify the rotten apple? And Liver-in-Cube: Promoting Precision Medicine for Liver Cancer Patients were explored and discussed.

In the basic science session on cutting edge spatially-resolved Omics and Proteomics in Liver Transplantation, many aspects of applications of proteomics and multi-omics technology were shared. David Al-adra spoke on how omics/spatial transcriptomics can predict clinical outcomes in liver transplantation and this topic was further enhanced by Sonya MacParland from Toronto on high-definition spatial transcriptomics in transplantation. Anne Peters from Cincinnati define the T cell transcriptional landscape in pediatric liver transplant rejection at single cell resolution and finally Sergio Duerte from Florida spoke on multi-omic characterization of cellular reprogramming in HBV and HCC to complete the session.

### Meet the journal editors session

For the first time, ILTS 2025 in Singapore invited the Editor-in-Chiefs (EIC) from various journals including Liver Transplantation and American Journal of Transplantation, as well as past EIC for JAMA to share their experience with the participants. Entitled “Navigating the Future of Publishing in Liver Transplantation: Challenges and Innovations” as the theme for the session, the 3 EICs spoke on “The Rise of AI in Submissions: Navigating Authorship, Ethics, and Editorial Policy”, “Beyond the Impact Factor: What Really Matters in Journal Influence?” and “Peer Review in Crisis? Quality, Speed, and the Human Cost”. Dr Sandy Feng (USA) clarified ICMJE authorship standards—AI may aid manuscript preparation under human oversight but cannot be a co-author or participate in peer review. Dr Howard Bauchner (USA) acknowledged peer review's limits in detecting fraud whilst affirming its necessity, predicting a shift toward open access with responsibly regulated AI assisting reviewer burden. The session was extremely well attended with high value advice provided by the EICs to improve on the academic article submissions in the future.

### Leadership and innovation in liver transplantation: A debate across generations

This was an interesting section where we invited world leaders from across generations encompassing the 4 major pillars of liver transplantation, including Transplant Surgery, Transplant Hepatology, Transplant Anaesthesia and Transplant Basic Science, to debate on the current state and future perspectives of each of these areas in liver transplantation. In the Liver Transplant Surgery: Evolution vs. Revolution section, many questions were raised and debated, including “Are traditional liver transplant techniques still the gold standard, or should robotics and AI-assisted surgery take the lead?”, “Should we continue prioritizing technical mastery, or is innovation in organ allocation and machine perfusion the bigger game-changer?”, and “What is the future trend of xenotransplantation and artificial organs that may revolutionize organ transplant care for patients?”. Subsequently, the Transplant Hepatology focused on “The Role of the Hepatologist: Gatekeeper or Partner in Liver Transplantation?”.

The Transplant Anaesthesia session debated on “Should liver transplant anesthesia and critical care be driven by algorithms and AI-assisted decision-making, or does human expertise and intuition still outweigh technology?”, and “With extracorporeal organ support and novel monitoring tools, are anesthesiologists and intensivists becoming the primary decision-makers in transplant success?”. Lastly, the Basic Scientists session entitled: “Basic Science in Liver Transplantation: Is the Future Big Data or Benchwork?” had the two scientists across generations debated about: “Is big data analytics poised to replace benchwork in liver transplantation research, or are they complementary approaches?”, “Which method has led to more clinically relevant breakthroughs in liver transplantation: computational modeling or traditional experimental research?”, “Do *in vitro* and *in vivo* models remain indispensable, or will in silico approaches become the gold standard for liver transplant research?”, “Can big data approaches lead to truly personalized immunosuppressive regimens, or do we still need bench-based functional assays?”, and “Does single-cell transcriptomics and spatial genomics in liver transplantation bridge the gap between big data and benchwork, or does it introduce new complexities?”.

These interesting and exciting debates had entertained the audience and had opened up a whole new horizon for audience to ponder of these topics regarding the future of liver transplantation.

## Emerging research and clinical perspectives in 2025

Several cross-cutting themes at ILTS 2025 Singapore point to where the field is heading over the next three to five years. The perspectives below are drawn directly from the scientific programme and emerging data presented at the congress (Figure 5).

## Robotic surgery as an emerging clinical standard

The trajectory from “pioneering” to “mainstream” is now clearly underway in robotic donor hepatectomy. Multi-centre registry data involving 2,600 donors at 62 centres, combined with single-centre series and the A2ROBOT study, provide compelling safety evidence. The convergence of RDH with 3D imaging, ICG fluorescence, AI-guided navigation, and ultimately autonomous surgical platforms—as described by Dr Yee Lee Cheah—represents one of the most tangible translational trajectories in modern transplant surgery. The extension of MIS to recipients (LALT, robotic LT) and paediatric APOLT further broadens the surgical frontier (2, 3).

## Machine perfusion as a therapeutic platform

The most forward-looking theme of ILTS 2025 Singapore was the reframing of NMP from a preservation tool to an active therapeutic platform. FMN as a real-time biomarker (AUROC 0.94) enables objective graft assessment. Spatial transcriptomics applied during NMP identifies molecular signatures predictive of ischaemic cholangiopathy. Long-term preservation (14+ days) provides a window for genetic editing, immune conditioning, and ex-situ oncological treatment before implantation. Together, these advances position NMP as the substrate for next-generation graft repair and molecular characterisation (3).

## Artificial intelligence in clinical transplant practice

AI in LT is no longer aspirational—it is in clinical use and being rigorously evaluated. From waitlist mortality prediction (LightGBM/SHAP outperforming MELD) to HCC recurrence risk (HCC-LT-ProBot), to intraoperative guidance and operative video analysis, AI applications were presented across every stage of the transplant pathway. The SOTA lecture by Professor Dean Ho framed AI as “augmented intelligence”—a tool that enhances clinical judgement rather than replacing it. Prospective multi-centre validation remains the essential next step before widespread implementation (3).

## Xenotransplantation at the clinical threshold

Gene-modified pig-to-human liver xenotransplantation (Tao et al., Nature 2025) (5) and porcine liver xenograft survival in a human decedent model (Shaked et al.) (6) represent a qualitative shift in the field. The session at ILTS 2025—“Uncharted Territory: Addressing Challenges in Liver Xenotransplantation”—was frank about the remaining immunological and biochemical barriers. The emerging consensus is that porcine livers as a bridging device (rather than a definitive transplant) represents the most near-term achievable clinical application, pending regulatory pathway development.

## Spatial transcriptomics and molecular biomarkers

High-dimensional molecular profiling is entering clinical translation. Al-Adra et al.'s spatial transcriptomic characterisation of DCD livers during NMP, Emamaullee's mapping of T-cell landscapes in paediatric ALF, MacParland's high-definition spatial transcriptomics, and Peters’ single-cell T-cell landscape in paediatric rejection together define a new paradigm: the biological state of the graft can now be characterised at single-cell resolution during the transplant process. The CAID biomarkers and cfDNA copy number variation risk scores extend molecular precision to post-transplant monitoring (3).

## Responsible innovation, financial sustainability & global equity

A theme of responsible innovation pervaded the entire congress. The Business Symposium provided a sober counterweight to the scientific ambition: high costs of combined NMP strategies demand simplification; robotic programme economics require novel funding models; AI requires prospective validation before clinical deployment at scale. For the Asia-Pacific region, heterogeneous DCD frameworks, HDI-stratified donor safety gaps, and financial barriers to advanced technologies represent enduring challenges. The field appears to have acquired a productive scepticism—one that welcomes transformative technology whilst demanding sustainable, equitable implementation (3).

## Conclusion & reflections: A field at the inflection point

The 30th ILTS Annual Congress in Singapore will be remembered as a congress of convergence—one at which multiple technological revolutions arrived simultaneously at clinical maturity. Robotics, machine perfusion, AI, and translational molecular science are no longer isolated innovations but are increasingly intersecting: AI guides robotic surgical navigation; MP provides the substrate for transcriptomic biomarker discovery; organoid models illuminate immunological mechanisms that AI tools attempt to predict from clinical parameters. This convergence is the most significant meta-narrative of the 2025 congress. (Figure 5)

For the Asia-Pacific region specifically, the choice of Singapore as host was substantively appropriate. The region accounts for the largest global volume of LDLT, drives significant technical innovation, and is increasingly represented in the highest-impact transplant science. Yet HDI-stratified disparities in donor safety and failure-to-rescue rates, heterogeneous regulatory frameworks for deceased donation and DCD, and financial barriers to advanced technologies represent enduring challenges that no individual institution can address alone.

DCD liver transplantation across Asia merits particular attention. Outside mainland China—which operates one of the world's most active DCD liver programmes under category II and DBCD frameworks—DCD liver transplantation remains nascent across most of the region. The machine perfusion advances presented in Singapore—FMN-guided organ assessment, spatial transcriptomic characterisation during NMP, and long-term ex situ preservation—provide both the scientific rationale and the practical toolkit for cautious, evidence-based DCD programme development as legislative and ethical frameworks evolve across Asian jurisdictions.
